# Pure sarcomatous recurrence of clear cell renal carcinoma following radical nephrectomy and dendritic cell vaccination

**DOI:** 10.1590/S1516-31802006000300011

**Published:** 2006-05-04

**Authors:** Alberto Azoubel Antunes, Marcos Francisco Dall'Oglio, José Alexandre Marzagão Barbuto, Kátia Ramos Moreira Leite, Miguel Srougi

**Keywords:** Renal cell carcinoma, Recurrence, Sarcoma, Nephrectomy, Dendritic cells, Carcinoma de células renais, Recidiva, Sarcoma, Nefrectomia, Células dendríticas

## Abstract

**CONTEXT:**

Sarcomatous differentiation, which represents transformation to high-grade malignancy, can occur in all histogical types of renal malignancy.

**CASE REPORT:**

The authors report on the case of a 66-year-old woman with a right renal mass that was shown to be a clear cell carcinoma. She underwent radical nephrectomy and dendritic cell vaccination and, 3.5 years later, she developed retroperitoneal pure sarcomatous recurrence of the tumor.

The authors speculate that the vaccination could have played some role in this differentiation or selection of the sarcomatous component of the primary tumor.

## RESUMO

**CONTEXTO:**

Diferenciação sarcomatosa, que representa evolução para malignidade de alto grau, pode ocorrer em todos os tipos histológicos de câncer renal.

**RELATO DE CASO:**

Os autores relatam o caso de uma mulher de 66 anos, com uma massa no rim esquerdo que se revelou um carcinoma de células renais. Após 3,5 anos da nefrectomia radical seguida de vacinação com células dendríticas, a paciente desenvolveu uma recorrência sarcomatosa pura no retroperitônio.

Os autores especulam que a terapia com vacina de células dendríticas pode ter desempenhado algum papel na diferenciação ou seleção do componente sarcomatoso do tumor primário.

## INTRODUCTION

Sarcomatous differentiation can occur in all histological subtypes of renal malignancy and represents a transformation to high-grade malignancy. The reported incidence ranges from 0.7% to 13.2% of all renal cell carcinomas (RCC).^1,2^

Studies have demonstrated that the presence of a sarcomatous component is associated with poor prognosis, and that surgical resection alone may not affect the clinical course of many patients.^1,2^ Although adjuvant dendritic cell (DC) vaccination can be useful for some patients, many of the biological and immunological effects of these drugs on RCC are unknown.^3^

The authors report on the case of a 66-year-old woman with RCC with sarcomatous differentiation, in whom there was retroperitoneal pure sarcomatous recurrence of the tumor, 3.5 years after radical nephrectomy and dendritic cell vaccination.

## CASE REPORT

**Figure 1:**
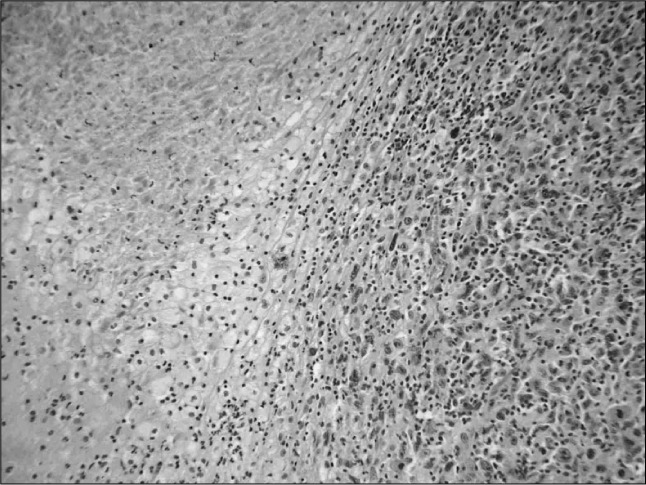
Primary tumor: renal cell carcinoma of clear cell type, extensively necrotic, presenting intense nuclear pleomorphism (Fuhrman grade IV) and mitotic activity.

A 66-year-old woman complained of mild pain in the right flank for a few months. The laboratory tests and chest x-ray were normal. Computed tomography (CT) showed a right renal mass measuring 9.8 x 9.2 cm and a 2.4 x 1.9 cm hepatic nodule that resembled hemangioma. She underwent right radical nephrectomy in January 2001. The pathology showed clear cell carcinoma with a sarcomatous component of nuclear grade IV, measuring 24 cm, with necrotic and hemorrhagic areas, perirenal fat involvement and extensive microvascular invasion (Figure 1).

With the patient's agreement, she was enrolled in a hybrid dendritic cell and tumor cell vaccination protocol at Hospital Sírio-Libanês one month after surgery. The vaccination was done at six-week intervals as described previously,^3^ and the patient was followed up by means of periodic laboratory tests and radiographic examinations.

Eight months later, abdominal CT showed a solid mass measuring 4 cm in the right renal bed. The patient was treated surgically and the pathology showed an undifferentiated large renal cell carcinoma with sarcomatous areas. No adjuvant therapy was given.

Thirty-four months later, the abdominal and pelvic CT again showed an extensive solid mass measuring 4.3 x 3.2 cm in the right renal bed, and another two solid masses measuring 5.0 x 3.5 cm and 3.5 x 2.5 cm, adjacent to the psoas muscle and anterior to the vena cava, respectively.

**Figure 2:**
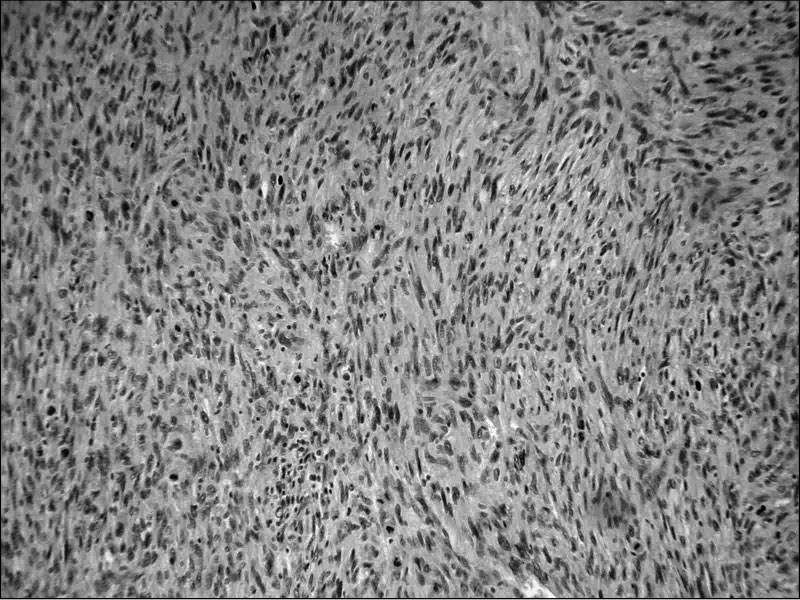
Recurrent tumor: sarcomatous carcinoma, resembling hemangiopericytoma.

**Figure 3:**
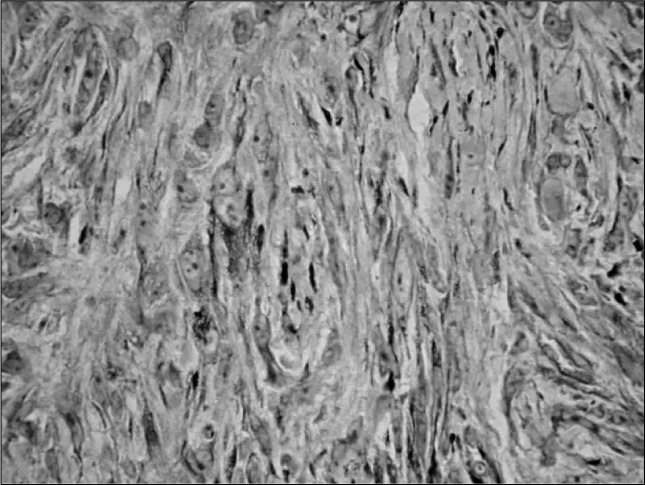
Immunohistochemistry showing positivity of the tumor cells for cytokeratin 18.

Another surgical resection was done and the pathology showed neoplasm consisting of pure spindle cell sarcoma resembling hemangiopericytoma, with no epithelial differentiation (Figure 2). The immunohistochemical analysis showed diffuse positive staining for cytokeratin 18 and vimentin (Figure 3). The patient was then sent for systemic chemo-therapy using ifosfamide and adriblastine and, after the fourth cycle, she is now presenting a 50% reduction of the residual tumor.

## DISCUSSION

Sarcomatous RCC is a locally aggressive and potentially metastatic disease.^1,2^ The two-year cancer-specific survival for patients with clear cell, chromophobe and papillary RCC with a sarcomatous component is 33%, 40% and 28% respectively, whereas the survival is 82%, 95% and 95% for patients with the same subtypes without a sarcomatous component.^1^ Surprisingly, despite the two retroperitoneal recurrences, our patient has shown good performance and no clinical sign of advanced disease, with more than three years of follow-up.

There is controversy regarding the degree of responsiveness of sarcomatous RCC to immunotherapy. Vaccination with dentritic cell-tumor cell hybrid seems to affect the natural history of advanced RCC, and a recent study has shown a 14% objective response for metastatic disease.^3^ Despite the enthusiasm for these strategies, more knowledge of dentritic cell biology and immune response boosting after vaccination is needed to improve the clinical responses.

To our knowledge, this is the first case reported of a pure sarcomatous transformation, in which the primary lesion had predominance of the epithelial component. Since primary renal sarcomas are very rare, accurate immunohisto-chemical investigation for epithelial elements must be done. The authors speculate that the vaccination could have played some role in this differentiation or selection of the sarcomatous component of the primary tumor.
